# Seroprevalence of SARS-CoV-2 Antibodies in Symptomatic Individuals Is Higher than in Persons Who Are at Increased Risk Exposure: The Results of the Single-Center, Prospective, Cross-Sectional Study

**DOI:** 10.3390/vaccines9060627

**Published:** 2021-06-09

**Authors:** Alexandr Zurochka, Maria Dobrinina, Vladimir Zurochka, Desheng Hu, Alexandr Solovyev, Liana Ryabova, Igor Kritsky, Roman Ibragimov, Alexey Sarapultsev

**Affiliations:** 1 School of Medical Biology, South Ural State University, 454080 Chelyabinsk, Russia; zurochkaav@susu.ru (A.Z.); zurochkava@susu.ru (V.Z.); 2 Institute of Immunology and Physiology, Ural Branch of the Russian Academy of Science, 620049 Ekaterinburg, Russia; mdobrynina87@gmail.com (M.D.); igor81218@gmail.com (I.K.); Ibragimovroman98@yandex.ru (R.I.); 3 Department of Integrated Traditional Chinese and Western Medicine, Union Hospital, Tongji Medical College, Huazhong University of Science and Technology, Wuhan 200092, China; desheng.hu@hust.edu.cn; 4 NPO National Medical Association for the Development of the Expert Activities in the Field of Laboratory Diagnostics “MedLabExpert”, 117042 Moscow, Russia; a.solovyev75@gmail.com; 5 LCC GMK MEDMA, 620102 Ekaterinburg, Russia; 6 Department of Propedeutics of Internal Diseases, South Ural State Medical University, 454092 Chelyabinsk, Russia; lianarabowa@rambler.ru; 7 Institute of Natural Sciences and Mathematics, Ural Federal University Named after the First President of Russia, 620026 Ekaterinburg, Russia

**Keywords:** antibody, COVID-19, focus groups, global health, high-risk groups, seroprevalence

## Abstract

The present study aimed to assess antibody seropositivity prevalence among symptomatic individuals and individuals with a high risk of occupational exposure to SARS-CoV-2. Participants from Chelyabinsk (Russian Federation) who were at an increased risk of exposure to SARS-CoV-2 (high-risk group, *n* = 1091) and participants who either had symptoms consistent with COVID-19 or were suspected to have experienced COVID-19 in the past (symptomatic group, *n* = 692) were enrolled between 28 September and 30 December 2020. Blood samples were tested by enzyme-linked immunosorbent assay D-5501 SARS-Cov-2-IgG-EIA-BEST and D-5502 SARS-Cov-2-IgM-EIA-BEST (AO Vector-Best, Novosibirsk, Russia). The overall seropositivity rate was 28.33–28.53%. SARS-CoV-2 antibodies were detected in 17.23% (adjusted prevalence of 17.17–17.29%) of participants in the high-risk and 45.95% (adjusted prevalence of 45.91–46.24%) in the symptomatic group. Higher IgG and IgM titers were observed in women compared to men, as well as in participants in the symptomatic group compared to those in the high-risk group. The results indicate that the seroprevalence among residents in several Russian regions is low (28.38%) and inadequate to provide herd immunity. The lower seroprevalence among participants in the high-risk group may be attributed to the enforcement of healthcare protocols and the use of adequate personal protective equipment.

## 1. Introduction

The coronavirus disease 2019 (COVID-19) pandemic has spread rapidly to more than 180 countries worldwide, resulting in high levels of morbidity and mortality. As of 9 May 2021, nearly 166 million COVID-19 cases and 3449 million deaths have been reported [1].

The clinical manifestations of COVID-19 can range from an asymptomatic/mild disease to severe disease with acute respiratory tract infections. Data from the meta-analysis suggest that the pooled prevalence of asymptomatic COVID-19 is about 48% and is higher in females than in males [2]. With that, because of the absence of symptoms and complaints, asymptomatic COVID-19 carriers can escape detection from the health system and, thus, are challenging for the implementation of preventive measures and infection control [2]. Thus, as the majority of currently available data are restricted to symptomatic patients with laboratory-confirmed COVID-19, the extent of the pandemic may be underestimated, and the virus may have a great potential for silent spread through the population [3,4]. Moreover, a recent meta-analysis reported a pooled ratio of serologically-detected infections to virologically-confirmed cases of 7.7; that is, for each confirmed case of severe acute respiratory syndrome coronavirus 2 (SARS-COV-2) infection, at least six infections remain undetected by current surveillance systems [5].

While CDC does not recommend serology testing to diagnose the current infection [6], its utility and importance for public health should be more emphasized in the COVID-19 pandemic [7]. As the duration of immunity to SARS-CoV-2 dictates the overall course of the pandemic and can also affect post-pandemic dynamics [8], serological studies are urgently needed [9]. The results of such studies can facilitate the assessment of infection spread, infection fatality rates, level of herd immunity, and the impact of interventions [5]. Public health decision-making would especially benefit from data pertaining to seroprevalence among individuals in occupations with a high risk of exposure to SARS-CoV-2 due to frequent social interactions (e.g., service-sector employees) [10].

The seroconversion of specific SARS-CoV-2 IgG/IgM antibodies can start as early as 4 days [11], with the median time of 7–8 days [12], after the onset of illness, and both IgG and IgM titers plateau within 6 days after seroconversion [13]. Most patients have neutralizing titers on days 14–20 with great titer variability [14]. The duration of the positivity rate exceeding 80% is about seven weeks for IgM and about 3–6 months for IgG [15]. According to the current data, the prevalence and dynamic characteristics of SARS-CoV-2 IgG/IgM antibodies are affected by age, sex, and disease severity [16]; however, the research population studied is still not comprehensive and that causes the discrepancies in results among the studies [14].

A considerable amount of literature has been published on the impact of the pandemic on healthcare workers [17], while the studies investigating other essential workers with direct customer exposure are limited [18,19]. With that, the elevated risk of infection was not limited to healthcare workers, and other high-risk occupations being affected during the pandemic comprised almost half of local transmission and the majority of the possible work-related cases [10,20,21].

Although the COVID-19 outbreak in Russia started later compared to many neighboring European countries, Russia is currently among the six countries with the highest number of confirmed COVID-19 cases, as of 9 May 2021 [22,23]. Limited data suggest that the seroprevalence of SARS-CoV-2 infection in Russia was approximately 9–10% in May–June 2020 [24], reaching 19.6–31.3% [25,26,27] by the end of the year. At present, there are no data for seropositivity rates in specific cohorts, such as those with a high risk of occupational exposure to SARS-CoV-2. Moreover, because of the high risk for infection spread by persons with an asymptomatic form of the disease, serological studies should include those from high-risk groups even without the symptoms.

Thus, the present pilot study aimed to assess and compare antibody seropositivity prevalence rates among symptomatic individuals and individuals in occupations with a high risk of exposure to SARS-CoV-2.

## 2. Materials and Methods

### 2.1. Location of the Study

Chelyabinsk is the seventh-largest city in Russia with a population of approximately 1.3 million. The city has a humid continental climate (Köppen: Dfb): the average temperature in January is −14 °C/6.6 °F. and 19 °C/66.7 °F in July. The first case in the city was registered on 21 March 2020, and 58,380 cases with 1417 deaths from COVID-19 were reported as of 24 April 2021.

### 2.2. Study Design, Population, and Sampling

In this cross-sectional study, we assessed the prevalence of SARS-CoV-2 infection via serological testing for anti-SARS-CoV-2 antibodies. A total of 1300 persons, who were at an increased risk of exposure to SARS-CoV-2 (i.e., healthcare workers, education staff, and supermarket employees), were invited to participate in a free employer-sponsored SARS-CoV-2 serology assessment between 28 September 2020 and 30 December 2020.

This study excluded patients younger than 18 years with a history of coronary heart disease, pre-excitation syndromes, motor impairments (cerebral palsy and epilepsy), with pacemakers, drug addicts, and dialysis patients.

One thousand and ninety-one persons were enrolled in the study and comprised the “high-risk” group; 209 persons did not agree to participate or did not attend the hospital for a scheduled appointment. Individuals were excluded from this group if they reported symptoms of COVID-19 or other acute respiratory virus infections on the day of the appointment.

Six hundred and ninety-two persons who had symptoms, reported having symptoms consistent with COVID-19 or other acute respiratory virus infections (fever, muscle pain, tiredness, headache, cough, sore throat, new loss of taste or smell, and a blocked nose), were suspected to have COVID-19 infection in the past, or had contact with COVID-19 infected persons and self-reported for enrolment in the study comprised the “symptomatic/contact group”.

### 2.3. Ethical Permission

The study proposal and protocol were approved by the ethics committee of the Institute of Health “DoctorLab” (1 July 2020). Reporting was in accordance with the Strengthening the Reporting of Observational Studies in Epidemiology guidelines. A representative of the research team approached the individuals by phone to ask if they would be willing to have a research coordinator speak to them about the study. If yes, the coordinator spoke with them, described the study (risks/benefits, voluntary participation, and procedures). Individuals were given adequate time to reflect on the information, had any questions answered, and gave free and voluntary consent. Patient consent forms were distributed to the participants at reception areas of the Institute of Health “DoctorLab” (LLC “DoctorLab”).

### 2.4. Laboratory Tests

Peripheral blood was collected by venipuncture in BD Vacutainer^®^ SST™ Tubes containing spray-coated silica and a polymer gel for serum separation (BD Biosciences, San Jose, CA, USA) and centrifuged at 1500× *g* for 20 min. The obtained blood serum was used for the IgM and IgG detection on the day of venipuncture. Serum samples were tested by enzyme-linked immunosorbent assay (ELISA) D-5501 SARS-Cov-2-IgG-EIA-BEST and D-5502 SARS-Cov-2-IgM-EIA-BEST (AO Vector-Best, Novosibirsk, Russia). All samples were tested in duplicate. The test was performed according to the manufacturer’s instructions [28].

The method of determination is based on a two-stage “indirect” version of ELISA. At the first stage of the analysis, the specific antibodies (IgG or IgM) contained in the test samples bind to the recombinant SARS-CoV-2 antigen immobilized on the surface of the plate wells—receptor-binding domain (RBD) of glycoprotein S (Spike, S-protein). At the second stage, the conjugate of monoclonal antibodies to human IgG (IgM) with horseradish peroxidase interacts with antigen–IgG (antigen–IgM) complexes. During incubation (25 min) with a tetramethylbenzidine solution, the solution stains in the wells contained the formed antigen–IgG-conjugate complexes. After stopping the reaction by addition of the stop solution (1 N H_2_SO_4_), absorbance at 450 nm with a 620-nm reference was measured in an ELISA plate reader. The intensity of the staining is proportional to the concentration of IgG (IgM) to SARS-CoV-2 in the analyzed sample. The total procedure requires 10 μL of plasma and the duration of the assay is about 2 h.

The sensitivity of the SARS-Cov-2-IgG-EIA-BEST is 71.6% during the initial stages of antibody production and 100% in later stages; the sensitivity of SARS-Cov-2-IgM-EIA-BEST is 82% and 95.4%, respectively [29]. The specificity of the test system used is 99.72–99.93% [29].

### 2.5. Adjusting Prevalence Estimates

The adjusted prevalence was estimated using the following formula according to Sempos and Tian [30]. The adjusted prevalence was calculated to avoid the test kit errors and to harmonize results over time and place [30].

(1) $$\mathrm{adjusted}\;\mathrm{prevalence}\;=\frac{\mathrm{crude}\;\mathrm{prevalence}\;+\;\mathrm{specificity}\;-1}{\mathrm{sensitivity}+\mathrm{specificity}-1}$$

### 2.6. Positivity Coefficient (CP) Calculations

Positivity coefficient (CP) calculations to display the antibody content were carried out following the manufacturer’s instructions [31]. CP shows how many times the concentration of antibodies exceeds the threshold value.

For this, the arithmetic mean values of optical density in the wells with a negative control sample (ODaverage. K^−^) were calculated. The results were accounted for if the following conditions were met—the average OD value in the well with K was not more than 0.2—OD value in the well with K^+^ was not less than 0.5.

On the next step, the critical value of optical density (ODcrit.) was calculated by the formula:

ODcritical = ODaverageK + 0.2.(2)

CP was calculated using the formula:

(3) CP = ODsample/ODcritical

where ODsample is the OD value in a well with control or analyzed sample.

The test results were considered positive if CP was equal or more than 1.1, negative—if CP was less than 0.8, doubtful—if the results were between 0.8 and 1.1.

### 2.7. Statistical Analysis

Data management and analysis were carried out using software R 3.1.1 12 (R Foundation for Statistical Computing, Vienna, Austria) and Microsoft Excel version 14.0. Since all selected groups of the general sample had an abnormal distribution (Shapiro–Wilk test < 0.05), the statistical criteria chosen for the calculations were nonparametric. To study the correlation between the age of patients and the concentration of immunoglobulins, the Spearman rank test was chosen. The Kruskal–Wallis rank test was used to compare the concentration of immunoglobulins between men and women and between individuals from the “high-risk” and “symptomatic” groups.

## 3. Results

Samples from 1101 men and 682 women with an average age of 39 years were investigated (Table 1). Of the 1091 persons who composed the “high-risk” group, 310 (28.41%) were women with an average age of 47.79 years and 781 (71.59%) were men (average age 39.91 years). Additionally, 372 (53.76%) women with an average age of 41.39 years and 320 (46.24%) men (average age 39.67 years) comprised the “symptomatic” group.

Blood samples were tested for the presence of both IgM and IgG with the above-mentioned ELISA kits. The detailed distribution of the seropositivity rates is presented in Table 2.

### 3.1. Seropositivity Rates for Joint Detection of IgG and IgM in Persons of High-Risk and Symptomatic Groups

Among the 1091 persons from the “high-risk” group, positive results for the presence of SARS-CoV-2 antibodies were detected in 17.23% of cases (IgG was detected in 4.49%, IgM—in 2.38%, both IgG and IgM—in 10.36% of cases), doubtful results—in 0.73% of cases (Table 2).

Among the 692 persons from the “symptomatic” group, positive results for the presence of SARS-CoV-2 antibodies were detected in 45.95% of cases (IgG was detected in 9.39%, IgM—in 4.48%, both IgG and IgM—in 32.08% of cases), doubtful results—in 1.01% of cases.

The crude seropositivity rate in the total sample was 28.38% (IgG were detected in 6.4%, IgM—in 3.2%, both IgG and IgM—in 18.78% of cases); doubtful results were obtained in 0.84% (Table 2). With that, the crude seroprevalence of SARS-CoV-2 antibodies was significantly higher in women (37.4%) than in men (22.89%). Thus, IgG were detected in 32.99% of women and 20.35% of men, IgM—in 28.45% of women and 17.98% of men, both IgG and IgM—in 24.19% of women and 15.44 of men.

### 3.2. Adjusted Seropositivity Rates for Joint Detection of IgG and IgM in Persons of High-Risk and Symptomatic Groups

The measurement errors of tests can result in biased prevalence estimates, and thus, the adjusted seropositivity rates were calculated [30] depending on the possible stage of infection when the samples were taken (early stages of infection, when the sensitivity of the tests was low, or the peak of infection, when sensitivity of the tests reached 100%) (Table 3).

According to the conducted calculations, the adjusted seropositivity in the total sample was 28.33–28.53% (for IgG-positive, 6.16–8.61%; for IgM-positive, 3.07–3.57%; both IgG and IgM-positive—18.73–18.87%).

Adjusted seropositivity was significantly higher in the “symptomatic” group—45.91–46.24% compared to the values (17.17–17.29%) of the “high-risk” group. Seropositivity for two immunoglobulins (the presence of IgM and IgG to SARS-CoV-2) was 10.29–10.37% in the “high-risk” group and 32.03–32.26% in the “symptomatic” group. Seropositivity of individual IgM-positive and IgG-positive patients from the “high-risk” group was 2.21–2.57% and 4.25–5.94%, respectively. For the “symptomatic” group, a similar calculation of seropositivity revealed the higher values: 9.17–12.81% for IgG-positive and 4.42–5.14% for IgM.

Adjusted seroprevalence of antibodies to SARS-CoV-2 in women was 45.91–46.24% (for IgG-positive, 8.57–11.98%; for IgM-positive, 4.17–4.86%; both IgG and IgM-positive—24.14–24.31%) and in men 17.17–17.29% (for IgG-positive, 4.66–6.52%; for IgM-positive, 2.38–2.77%; both IgG and IgM-positive—15.38–15.49%).

### 3.3. Antibody Content

The average content of IgM antibodies in the total sample was 1.59, and the average content of IgG was 3.17.

According to the Spearmen test, the statistically significant positive relationship was found between age and IgM levels (S = 852,570,000, rho = 0.084, *p*-value = 0.0004) and between age and IgG levels (S = 860,350,000, rho = 0.075, *p*-value = 0.015). A statistically significant positive relationship was also found between the IgG and IgM levels (S = 328,150,000, rho = 0.65, *p*-value < 0.0001).

**Figure 1:**
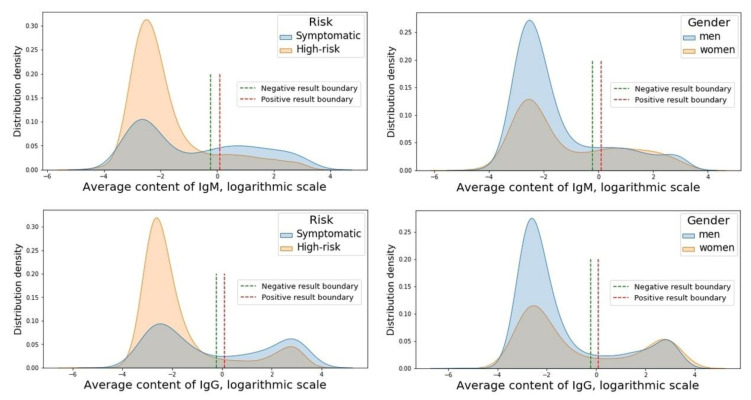
Distribution of IgM and IgG content with two peaks on both sides of the boundaries of the reference interval among persons from the symptomatic and high-risk groups and men and women.

According to the analysis, the bimodal distribution of IgM and IgG content with two peaks on both sides of the boundaries of the reference interval among men and women and persons from the symptomatic and high-risk groups (Figure 1) was present.

According to the Mann–Whitney test, the higher IgG and IgM levels were observed in women compared with men (for IgM-Mann–Whitney U-test = 3.476, *p*-value = 0.0005, ES = 0.11, Power = 0.37, Sample size = 4502; for IgG-Mann–Whitney U-test = −5.586, *p*-value ≤ 0.0001, ES = 0.33, Power = 0.99, Sample size = 502) and subjects from the symptomatic group compared with those from the high-risk group (for IgM-Mann–Whitney U-test = 8.490, *p*-value < 0.0001, ES = 0.46, Power = 0.99, Sample size = 260; for IgG-Mann–Whitney U-test = 11.975, *p*-value < 0.0001, ES = 0.44, Power = 0.99, Sample size = 284).

## 4. Discussion

COVID-19 is currently the top public health concern worldwide. It is estimated that approximately 97% of the world’s population is susceptible to SARS-CoV-2 [14]. Previous studies have indicated that the overall seroprevalence varies widely across different countries and regions, with a higher seropositivity prevalence being observed in locations of early outbreaks, as well as countries with higher income levels and human development index scores [10,32,33].

During the pandemic, the assessment of IgM and IgG has been used to diagnose COVID-19, allowing the evaluation of not only the cumulative prevalence of SARS-CoV-2 infection but also the monitoring of seroconversion at the individual and community levels [32,34]. The overall seropositivity estimate in the present study (28.33–28.53%) is similar to that reported from the general population of the Ural Federal District of Russia (24.5–31.3%), which comprises six federal districts: the Tyumen Region, Chelyabinsk Oblast, and Republic of Tatarstan [25,26]. However, our observed seroprevalence is significantly higher than the earlier estimate of 9–10.8% from May to June [24]; this reflects the gradual development of herd immunity and that SARS-CoV-2 continues to spread through the population, resulting in more symptomatic infections and, thus, more seropositive individuals.

The present study was conducted among persons in occupations with a high risk of exposure to SARS-CoV-2 [35]. It is considered that these persons experience a potentially higher SARS-CoV-2 exposure risk due to the nature of their job than the general population [10,20,21]. Thus, according to the study of F-Y Lan et al. (2020), employees with direct customer exposure were five times more likely to test positive for SARS-CoV-2 [18], while the seroprevalence of COVID-19 amongst police officers was at least 3.4 higher than in the general population [36]. Nevertheless, some recent data suggest that the seroprevalence of SARS-CoV-2 antibodies in persons from high-risk groups is similar [3] or only marginally higher [10,37] compared to that in the general population, depending on regional variation in COVID-19 incidence. Based on this, we initially assumed that the seroprevalence levels in the high-risk group would be near the upper limit of the general population. However, SARS-CoV-2 antibodies were only detected in 28.38% (adjusted prevalence 28.33–28.53%) of cases, which is significantly lower than the seroprevalence of the general population in neighboring regions [26,27]. The lower seroprevalence may be attributed to greater availability and enforcement of healthcare protocols, as well as the use of adequate personal protective equipment.

The prevalence (45.95%) and levels of SARS-CoV-2 antibodies in participants from the symptomatic group were significantly higher than those in the high-risk group. These results match other studies [16] and are similar to those reported by Naaber et al. (2020), who found that the positivity rate in asymptomatic COVID-19 cases was approximately two times lower compared to polysymptomatic cases; furthermore, patients with more symptoms usually had a higher positivity rate and antibody level [38].

Existing evidence suggests that seroprevalence worldwide is equivalent between sexes [10,17]. Moreover, according to the recent study of C Luo et al. (2021), SARS-CoV-2 IgG/IgM dynamic is mainly affected by age and disease severity, not sex [16]. However, the present study found a higher seroprevalence in women (37.4%) than in men (17.17–22.89%); these values are similar to those reported from neighboring regions of Russia [25,26]. There are several possible explanations for this result. This may be attributable to many factors, including similar age and sex distributions, employment structures, and/or cultural practices in Russia. Although, this finding may be explained by the fact that the decline positive rate of IgG/IgM antibodies is lower and the average titer of IgG/IgM antibodies is relatively higher in females from disease onset to 60 days [16,39].

Thus, consistent with the literature, this research found that antibody levels were higher in women compared to men, as well as in older versus younger participants [16,37,39,40]. Data from several studies suggest that the immune response to most pathogen vaccines in men is lower than that in women [41,42]. Estrogen and testosterone promote and suppress, respectively, the innate and adaptive immune systems [40]. Within the innate immune system, estrogen regulates innate myeloid (monocytes, dendric cells, neutrophils) and lymphoid cells and promotes type I IFN synthesis [43,44]. In turn, within the adaptive immune system, the higher numbers of CD4+ Helper T cells, more robust CD8+ (cytotoxic) T cells cytotoxic activity, and higher B cell production of immunoglobulin are observed in women compared to men [45]. Thus, women have an increased capacity to mount greater magnitudes of immune responses against the infection compared to men [45], and that may underlie the different outcomes between sexes [39,46].

However, the contemporary clinical data are rather controversial, and there is no general agreement about the impact of sex on antibody generation and prognosis in SARS-CoV-2 infection [16,47,48,49,50,51,52]. While several studies have reported higher levels of antibodies in women [39,48], other studies have reported equivalent levels between men and women [16,39], as well as higher levels in men [40,49,50,51]. Thus, further research should be undertaken to investigate the impact of sex and gender on immune response and associated adverse COVID-19 outcomes and to tailor the potential treatment according to sex and gender [45,51,52].

The obtained results are in accord with recent studies indicating that average IgG/IgM antibody levels were higher in old ages [16,53]. These results are likely to be related to an increased baseline level of proinflammatory cytokines associated with such comorbidities as obesity, hypertension, or diabetes, which are common in the elderly and could have a stimulatory effect on the SARS-CoV-2 humoral response [53,54].

## 5. Conclusions

The herd immunity threshold for SARS-CoV-2 is approximately 67% [55]. The results of this study indicate that the seroprevalence in Russia is relatively low and inadequate for herd immunity. Therefore, we emphasize the importance of maintaining current public health measures and intensifying vaccination efforts to keep the outbreak under control.

This study had several limitations due to the scale of the COVID-19 pandemic. First, this study was limited to a single city. Therefore, the results may not be generalizable to other regions that are more geographically diverse. Furthermore, due to the regulation rules, persons aged >65 years were not permitted to work during the pandemic; this may have affected the seropositivity rates observed in this study. Second, as random sampling was not used, the estimated seroprevalence was subject to potential sampling bias. Third, samples collected from infected individuals outside the antibody response time window and low diagnostic rates of the used commercial test systems in the initial phase of infection may have yielded false-negative results; therefore, the observed seroprevalence in our study may have underestimated the true prevalence rate of COVID-19. Fourth, we did not evaluate dynamic changes in antibody titers in infected individuals over time, and depending on the sampling time, there might be a higher incidence of seropositive individuals in the symptomatic group. Fifth, the prevalence estimates may change with new information on the accuracy of the test kits that we used. Sixth, since the majority of COVID-19 patients are either asymptomatic or have only a few mild symptoms, the sensitivity of the antibody tests in the general population may be lower; this may affect the reliability of antibody-based epidemiological studies [20].

## Figures and Tables

**Table 1:** Demographic characteristics of the study participants.

Risk Group	Gender	Count	Prevalence within the Group	Prevalence	Mean Age
High-risk	Men	781	72%	44%	36.91
	Women	310	28%	17%	41.79
Symptomatic	Men	320	46%	18%	39.67
	Women	372	54%	21%	41.39

**Table 2:** Seropositivity rates for joint detection of IgG and IgM in persons of high-risk and symptomatic groups.

Variant	Presence [+] or Absence [−] of Igs	Group	*n*	Prevalence in the Group	Prevalence in the Study Population
	Men vs. Women				
1	IgM [−] and IgG [−]	women	423	62.02%	23.72%
		men	839	76.20%	47.06%
2	IgM [−] and IgG [+]	women	41	6.01%	2.30%
		men	44	4.00%	2.47%
3	IgM [−] and IgG [*]	women	3	0.44%	0.17%
		men	3	0.27%	0.17%
4	IgM [+] and IgG [−]	women	21	3.08%	1.18%
		men	25	2.27%	1.40%
5	IgM [+] and IgG [+]	women	165	24.19%	9.25%
		men	170	15.44%	9.53%
6	IgM [+] and IgG [*]	women	8	1.17%	0.45%
		men	3	0.27%	0.17%
7	IgM [*] and IgG [−]	women	2	0.29%	0.11%
		men	7	0.64%	0.39%
8	IgM [*] and IgG [+]	women	19	2.79%	1.07%
		men	10	0.91%	0.56%
	High−risk vs. Symptomatic				
1	IgM [−] and IgG [−]	high−risk	895	82.03%	50.20%
		symptomatic	367	53.03%	20.58%
2	IgM [−] and IgG [+]	high−risk	45	4.12%	2.52%
		symptomatic	40	5.78%	2.24%
3	IgM [−] and IgG [*]	high−risk	3	0.27%	0.17%
		symptomatic	3	0.43%	0.17%
4	IgM [+] and IgG [−]	high−risk	21	1.92%	1.18%
		symptomatic	25	3.61%	1.40%
5	IgM [+] and IgG [+]	high−risk	113	10.36%	6.34%
		symptomatic	222	32.08%	12.45%
6	IgM [+] and IgG [*]	high−risk	5	0.46%	0.28%
		symptomatic	6	0.87%	0.34%
7	IgM [*] and IgG [−]	high−risk	5	0.46%	0.28%
		symptomatic	4	0.58%	0.22%
8	IgM [*] and IgG [+]	high−risk	4	0.37%	0.22%
		symptomatic	25	3.61%	1.40%

Note: [+]—positive results; [−]—negative results; [*]—doubtful results. The overall seropositivity was a calculated sum of variants [2 + 8] (IgG)/[4 − 6] (IgM)/[5] (IgM + IgG).

**Table 3:** Calculation of adjusted seropositivity in men and women using open data on sensitivity and specificity.

Gender/Risk Group	6–12 Days *			13–20 Days *		
	Adj. Prev.	Cr. Prev.	Adj. Count	Adj. Prev.	Cr. Prev.	Adj. Count
IgM and IgG positive						
All patients	18.87%	18.79%	336	18.73%	18.79%	334
Women	24.31%	24.19%	166	24.14%	24.19%	165
Men	15.49%	15.44%	171	15.38%	15.44%	169
High-risk	10.37%	10.36%	113	10.29%	10.36%	113
Symptomatic	32.26%	32.08%	223	32.03%	32.08%	218
IgG positive						
All patients	8.61%	6.39%	153	6.16%	6.39%	110
Women	11.98%	8.80%	82	8.57%	8.80%	58
Men	6.52%	4.90%	72	4.66%	4.90%	51
High-risk	5.94%	4.49%	65	4.25%	4.49%	47
Symptomatic	12.81%	9.39%	89	9.17%	9.39%	63
IgM positive						
All patients	3.57%	3.20%	64	3.07%	3.20%	55
Women	4.86%	4.25%	33	4.17%	4.25%	28
Men	2.77%	2.54%	30	2.38%	2.54%	26
High-risk	2.57%	2.38%	28	2.21%	2.38%	24
Symptomatic	5.14%	4.48%	36	4.42%	4.48%	30

Note: *—from the possible 1st day of the disease; Cr. Prev.—crude prevalence; Adj. prev.—adjusted prevalence [30].

## Data Availability

The datasets analyzed during the current study are available from the corresponding author on reasonable request as they contain information on the gender, age, work experience, and places of work of the respondents.
